# Blastomeres of 8-cell mouse embryos differ in their ability to generate embryonic stem cells and produce lines with different transcriptional signatures

**DOI:** 10.3389/fcell.2023.1274660

**Published:** 2023-10-09

**Authors:** Sandra Alonso-Alonso, Anna Esteve-Codina, Beatriz Martin-Mur, Lucia Álvarez-González, Aurora Ruiz-Herrera, Josep Santaló, Elena Ibáñez

**Affiliations:** ^1^ Genome Integrity and Reproductive Biology Group, Departament de Biologia Cel·lular, Fisiologia i Immunologia, Facultat de Biociències, Universitat Autònoma de Barcelona, Barcelona, Spain; ^2^ CNAG-CRG, Centre for Genomic Regulation, Barcelona Institute of Science and Technology (BIST), Barcelona, Spain; ^3^ Universitat Pompeu Fabra (UPF), Barcelona, Spain; ^4^ Genome Integrity and Instability Group, Institut de Biotecnologia i Biomedicina, Universitat Autònoma de Barcelona, Barcelona, Spain

**Keywords:** ESCs, epiblast, cell fate, pluripotency, developmental potential

## Abstract

Embryonic stem cell (ESC) derivation from single blastomeres of 8-cell mouse embryos results in lower derivation rates than that from whole blastocysts, raising a biological question about the developmental potential of sister blastomeres. We aimed to assess the ability of 8-cell blastomeres to produce epiblast cells and ESC lines after isolation, and the properties of the resulting lines. Our results revealed unequal competence among sister blastomeres to produce ESC lines. At least half of the blastomeres possess a lower potential to generate ESCs, although culture conditions and blastomeres plasticity can redirect their non-pluripotent fate towards the epiblast lineage, allowing us to generate up to seven lines from the same embryo. Lines originated from the same embryo segregated into two groups according to their transcriptional signatures. While the expression of genes related to pluripotency and development was higher in one group, no differences were found in their trilineage differentiation ability. These results may help to improve our understanding of the ESC derivation process from single blastomeres and cell fate determination in the preimplantation mouse embryos.

## Introduction

Embryonic stem cells (ESC), traditionally derived from the inner cell mass (ICM) of blastocysts (Evans and Kaufman, 1981; Martin, 1981), can also be obtained from single blastomeres (Delhaise et al., 1996; Chung et al., 2006; Klimanskaya et al., 2006; Wakayama et al., 2007; González et al., 2010). This approach allows the generation of ESCs from a single cell, while the remaining embryo maintains its viability (Chung et al., 2008), thus enabling the generation of ESCs from valuable animal and human embryos without the need to destroy them. Alternatively, the separate use of all blastomeres for ESCs derivation, while leading to the destruction of that particular embryo, could allow the generation of multiple ESCs lines from a single embryo, thereby reducing the total number of embryos required to produce various ESCs lines. On the other hand, individual blastomeres from 8-cell embryos have been used to obtain mouse ESCs (mESCs) lines with an expanded potential to generate descendants of both trophectoderm (TE) and ICM. Remarkably, upon introduction into a blastocyst, these expanded potential mESCs lines can contribute both to the embryo proper and to extra-embryonic tissues (Yang et al., 2017).

Currently, the main limitation of using single blastomeres as a source of ESCs is the decreased derivation rate compared to the use of whole embryos, particularly when blastomeres of 8-cell embryos are used. Because ESCs are known to derive from the pluripotent epiblast (EPI) of the preimplantation embryo (Boroviak et al., 2014), this raises a significant biological question about the distribution of developmental potency among sister blastomeres of 8-cell embryos, that is, whether all sister blastomeres maintain their totipotent/pluripotent potential or whether some of the blastomeres are already committed to a non-pluripotent cell fate. In the first case, all blastomeres should be able to generate ESCs lines under the appropriate conditions and technical limitations of the experimental procedure could be the reason behind the failure of some blastomeres to produce ESCs. Therefore, efforts to improve culture conditions to maintain the inherent developmental potential of blastomeres after isolation could be pursued to improve derivation rates. In the second case, some blastomeres could be biologically unable to generate ESCs, therefore strategies other than improving culture conditions would be required to improve derivation rates. So far, up to three and five ESCs lines have been derived from sister blastomeres of human (Taei et al., 2013) and mouse (Hassani et al., 2014a) 8-cell embryos, respectively. However, the low number of embryos used in these studies, which were not aimed at resolving this question, is insufficient to reach a solid conclusion.

During preimplantation development, blastomeres gradually lose their developmental potential as cleavage progresses, and eventually differentiate into three separate lineages. It is widely accepted that the formation of the ICM and TE in the blastocyst is the first lineage segregation in the mammalian embryo (Cockburn and Rossant, 2010), followed by a second segregation where the outer cells of the ICM form the primitive endoderm (PE) and the EPI arises from the inner cells. TE and PE cells will give rise to the extraembryonic tissues whereas pluripotent EPI cells will generate all the cells of the embryo proper. It is still unclear at what developmental stage embryonic cells start to differ in their potential to differentiate into these multiple lineages, but mounting evidence suggests that a developmental bias may already be present in early cleavage-stage blastomeres. In this sense, recent studies have shown that sister blastomeres of 2-cell and 4-cell mouse embryos have unequal capacities to form EPI, PE and TE lineages (Casser et al., 2017; Krawczyk et al., 2021; Maemura et al., 2021). Multicolor lineage tracing has also revealed that sister blastomeres of intact 4-cell embryos display a bias in contributing to either TE or ICM (Tabansky et al., 2013). Additionally, interblastomeric molecular differences in transcriptional profiles, histone modifications and transcription factors kinetics, among others, have been detected as early as the 2-cell stage (reviewed in Bruce, 2013; Boiani et al., 2019; Toyooka, 2020; White and Plachta, 2020), although whether these differences are relevant for subsequent cell fate decisions remains an unresolved question.

Although heterogeneities among blastomeres in cleavage-stage embryos could play a role in cell fate commitment during normal development, it is known that preimplantation embryos are able to adapt to experimental perturbations such as the addition or removal of cells (Tarkowski and Wróblewska, 1967) because of their developmental plasticity. Therefore, even if a developmental bias exists among blastomeres in the intact early embryo, blastomere fate may not yet be irreversibly restricted at pre-compactional stages (Toyooka, 2020) and thus could be experimentally altered. For instance, the isolation of blastomeres from their normal environment could lead to changes in signaling pathways which, in turn, could generate transcriptional and epigenetic alterations, changing cell fate or removing the pre-existing developmental bias. Similarly, the addition of signaling modulators to the culture medium could alter blastomeres’ cell fate. In this sense, some studies with single blastomeres from 4-cell embryos suggest that their plasticity should allow all of them to generate ESCs upon being isolated from the embryo, regardless of whether their cell fate is predetermined in the intact embryo (Geens et al., 2009; González et al., 2011a). On the contrary, blastomeres from 8-cell embryos seem to lean towards a TE fate after isolation (Lorthongpanich et al., 2008), which could explain the low rates of mESCs derivation from these blastomeres (Wakayama et al., 2007; González et al., 2010). Nonetheless, short exposure of blastomeres to a chimeric E-cadherin (González et al., 2011b) or incubation in the presence of 2i (Ying et al., 2008) or R2i (Hassani et al., 2014b) cocktails can increase mESCs derivation rates (Hassani et al., 2014b; Alonso-Alonso et al., 2022).

The aim of this study was to determine whether, under optimized culture conditions that promote pluripotency maintenance and expansion of the EPI cell population, all blastomeres of the 8-cell stage mouse embryo are able to generate mESCs lines, and whether the lines originating from the same embryo have similar characteristics or whether they differ depending on the blastomere from which they originated. To this aim, we first examined the potential of individual sister blastomeres of 8-cell embryos to develop into mESCs lines and precursor cell aggregates containing pluripotent EPI cells. Next, we compared the transcriptional profile of the mESCs lines derived from blastomeres of the same 8-cell embryos and their ability to differentiate into cells of the three primary germ layers. Our findings will help to improve the generation of ESCs from single blastomeres and to better understand the developmental potential of single blastomeres of 8-cell stage embryos and the determination of cell fate during preimplantation embryo development.

## Material and methods

### Animal procedures

Mouse care and procedures were conducted according to the protocols approved by the Ethics Committee on Animal and Human Research of the Universitat Autònoma de Barcelona and by the *Departament d’Agricultura, Ramaderia, Pesca i Alimentació of the Generalitat de Catalunya* (permit numbers 4090 and 9995, respectively).

### Collection and biopsy of embryos

Mouse embryos were obtained from 6 to 12 weeks old B6CBAF1/J females (Charles River Laboratories). Females were induced to superovulate by intraperitoneal injection of 5 IU Pregnant Mare’s Serum Gonadotropin (MSD Animal Health) followed by the injection of 5 IU Human Chorionic Gonatropin (Divasa-Farmavic) 48 h later and mated with B6CBAF1/J males. Oviducts were flushed with HEPES-buffered KSOM medium (H-KSOM) (Biggers et al., 2000) 48 h later to collect 2-cell stage embryos, which were immediately cryopreserved following standard slow-freezing procedures (Costa-Borges et al., 2009) and stored in liquid N_2_ at −196°C. Cryopreservation allowed us to optimize the use of donor females and to work with the exact number of embryos required for each experimental replicate regardless of superovulation success.

When required, embryos were thawed (Costa-Borges et al., 2009), cultured in EmbryoMax^®^ KSOM Medium (Merck Millipore) under mineral oil (Sigma) at 37°C and 5% CO_2_, and biopsied when they reached the 8-cell stage, as previously reported (Alonso-Alonso et al., 2022). Embryo biopsy, instead of embryo disaggregation, was used to isolate blastomeres to minimize injury and avoid compromising their developmental ability. Only the embryos from which all blastomeres could be successfully isolated were used in subsequent experiments.

### Culture of single blastomeres for mESCs derivation

Single blastomeres were individually cultured in 50 μL drops of mESCs derivation medium that contained a feeder layer of mitomycin C-inactivated human foreskin fibroblasts (HFF-1; ATCC®SCRC-1041™), as previously reported (Alonso-Alonso et al., 2022).

Mouse ESC derivation medium (KSR + R2i) consisted of DMEM high glucose (BioWest) supplemented with 100 μM 2-β mercaptoethanol (Gibco), 1x non-essential amino acids (Gibco), 50 U/mL penicillin and 50 μg/mL streptomycin (Biowest), 20% KSR (Gibco ThermoFisher), 10^3^ U/mL leukemia inhibitory factor (LIF; Merck Millipore) and R2i inhibitor cocktail, a combination of MEK inhibitor PD0325901 (Axon Medchem; 1 μM) and TGFβ inhibitor SB431542 (Sigma, 10 µM). During the first week of culture, 0.1 mg/mL adrenocorticotropic hormone (ACTH; Prospec) was also added to the medium.

Blastomeres were cultured at 37°C and 5% CO_2_ for 7–10 days, and the medium was changed every 48 h until outgrowths were observed. After the first passage, the outgrowths were cultured in 4-well plates without ACTH. Putative mESCs lines were passaged once or twice a week using trypsin-EDTA (BioWest) and maintained in culture for at least six passages.

### Pluripotency characterization of mESCs lines

The stemness of all putative mESCs lines was assessed at the seventh passage by Alkaline Phosphatase Activity (ALP) staining and immunodetection of pluripotency and differentiation markers.

ALP activity was determined using a commercial kit (Sigma AB0300) and a previously reported protocol (Alonso-Alonso et al., 2022). Briefly, cells were fixed in 4% paraformaldehyde (PFA; Sigma Aldrich) for 1 min at room temperature (RT) and incubated for 10 min at RT in the dark in a 1:1 mixture of 5-bromine-4-chloride-3-inodyl phosphate and nitroblue tetrazolium. Cells were observed under a microscope and the pluripotency of the putative mESCs lines was determined based on the blue color of the colonies.

Immunofluorescence staining was performed both before and after spontaneous *in vitro* differentiation of the putative mESCs lines to confirm their pluripotency and differentiation potential, respectively. Spontaneous differentiation was achieved by culturing s lines in DMEM high glucose supplemented with 10% fetal bovine serum (FBS; BioWest) under feeder-free conditions for 10 days. Cells were fixed in 4% PFA for 20 min at RT, and blocked and permeabilized with a PBS solution containing 0.2% sodium azide (Sigma), 0.5% Triton X-100 (Sigma) and 3% goat serum (BioWest) for 30 min at 37°C. The fixed cells were incubated with primary antibodies overnight at 4°C, washed, and incubated with secondary antibodies for 2 h at RT.

Mouse monoclonal anti-OCT4 (Santa Cruz Sc-5279, 1:50 dilution), rabbit polyclonal anti-NANOG (Abcam ab80892, 1:100 dilution) and rabbit polyclonal anti-SOX2 (Merck Millipore ab5603, 1:200 dilution) antibodies were used to detect the pluripotency markers. Mouse monoclonal anti-Tubulin β3 (TUJ1; BioLegend 801201, 1:500 dilution), mouse monoclonal anti-*α* smooth muscle actin (αSMA; Sigma A5228, 1:200 dilution) and mouse monoclonal anti-alpha-fetoprotein (AFP; R&D Systems MAB1368, 1:50 dilution) antibodies were used to detect ectoderm, mesoderm, and endoderm differentiation markers, respectively. The secondary antibodies used were anti-mouse IgG Alexa Fluor 488 (Molecular Probes - Invitrogen A-21200, 1:500 dilution) for OCT4, TUJ1, αSMA and AFP, and anti-rabbit IgG Alexa Fluor 594 (Molecular Probes - Invitrogen A-11037, 1:500 dilution) for SOX2 and NANOG.

Finally, the cells were mounted with 10 μg/mL Hoechst 33258 (Molecular Probes, Invitrogen) diluted in Vectashield (Vector Laboratories) and analyzed using an epifluorescence microscope (Olympus BX61) and Cytovision software (Applied Imaging, Inc.).

### Culture of single blastomeres for cell aggregates formation

Single blastomeres were seeded on a monolayer of inactivated HFF (iHFF) in KSR + R2i medium, as detailed for the derivation of mESCs, and incubated at 37°C and 5% CO_2_ for 72 h to allow the formation of cell aggregates. Whole 8-cell embryos previously incubated in acidic Tyrode’s solution to remove the zona pellucida were also cultured under the same conditions and used as controls.

### Immunofluorescence staining of cell aggregates

Cell aggregates were fixed and immunostained following the same protocols described above for mESCs. Mouse monoclonal anti-OCT4 and rabbit polyclonal anti-NANOG were used as primary antibodies for the detection of ICM and EPI cells, respectively. The secondary antibodies used were anti-mouse IgG Alexa Fluor 488 for OCT4 and anti-rabbit IgG Alexa Fluor 594 for NANOG.

Finally, the cell aggregates were mounted with 10 μg/mL Hoechst 33258 diluted in Vectashield. The number of total cells (Hoechst staining), OCT4+ cells and NANOG + cells were counted using an epifluorescence microscope (Olympus BX61) with the Cytovision and ImageJ software.

### RNA-seq library preparation and sequencing

Feeder cells were removed from mESCs cultures using the protocol described by Sakai et al. (2011). Briefly, cells were trypsinized for 5 min, trypsin was inactivated by the addition of an equal volume of medium, and then the cell suspensions were transferred to a 0.1% gelatin-coated plate and incubated for 15 min at 37°C, 5% CO_2_. After this short incubation*,* feeder cells attached to the plates whereas mESCs remained in solution.

Total RNA was extracted from mESCs using the Maxwell RSC SimplyRNA Tissue Kit (Promega). Its concentration and quality were assessed using a NanoDrop spectrophotometer (ThermoFisher Scientific).

Two micrograms of total RNA obtained from each mESC line was sent to the Centre Nacional d’Anàlisi Genòmica (CNAG) in Barcelona (Spain) for RNA sequencing (RNA-seq). The samples were quantified using a Qubit^®^ RNA BR Assay kit (Thermo Fisher Scientific), and RNA integrity was estimated using an Agilent RNA 6000 Pico Bioanalyzer 2,100 Assay (Agilent). All the samples used in the study had RNA integrity numbers (RIN) above 9.

RNA-Seq libraries were prepared with the KAPA Stranded mRNA-Seq Illumina Platforms Kit (Roche) following the manufacturer´s recommendations. Briefly, 500 ng of total RNA was used for poly-A fraction enrichment with oligo-dT magnetic beads following mRNA fragmentation. The strand specificity was achieved during the second strand synthesis performed in the presence of dUTP instead of dTTP. The blunt-ended double stranded cDNA was 3′adenylated and Illumina platform compatible adaptors with unique dual indexes and unique molecular identifiers (Integrated DNA Technologies) were ligated. The ligation product was enriched using 15 PCR cycles. The size and quality of the libraries were assessed using a High-Sensitivity DNA Bioanalyzer assay (Agilent).

The libraries were sequenced on a NovaSeq 6,000 (Illumina) with a read length of 2 × 51 bp, following the manufacturer’s protocol for dual indexing. Image analysis, base calling and quality scoring of the run were processed using the manufacturer’s software Real Time Analysis (RTA 3.4.4).

### RNA-seq data processing and analysis

RNA-seq reads were mapped against the *Mus musculus* reference genome (GRCm39) using STAR software version 2.7.8a (Dobin et al., 2013) with ENCODE parameters. The percentage of uniquely mapped reads ranged from 86% to 88%, corresponding mostly to protein coding genes (average of 97%), and less than 1% on average were mapped to rRNA genes. Annotated genes were quantified with RSEM v1.3.0 (Li and Dewey, 2011) using the annotation file from GENCODE version M27 and default parameters.

Differential expression analysis was performed with the DESeq2 v1.18R package (Love et al., 2014) using a Wald test to compare the different embryos. Genes were considered differentially expressed (DE) with an adjusted *p*-value <0.05 and absolute fold change |FC| > 1.5.

Between-embryo variation was corrected from raw counts using the ComBat-seq function from the sva R package v3.40 (Zhang et al., 2020). Adjusted counts were regularized log transformed (rlog) using DESeq2. A weighted gene correlation network analysis (WGCNA) of genes with variance >0.05 was conducted with the WGCNA R package v1.69 (Langfelder and Horvath, 2008). Modules were detected using an automatic gene network construction function, lowering the sensitivity by setting the *deepSplit* parameter to 0. Eigengene values were calculated for each module, and module membership values were determined for each gene. The modules were characterized by Gene Ontology (GO) enrichment analysis using gProfileR v07.0 (Kolberg et al., 2020).

A Principal Component Analysis (PCA) plot was generated with the rlog transformed counts using the top 500 most variable genes and the ggplot2 R library (Wickham, 2009). Heatmaps with the top 50 DE genes were generated using scaled rlog-transformed counts with the pheatmap R package. Heatmaps with specific gene expression of core, naïve and primed pluripotency were generated using the pheatmap R package.

### Embryoid bodies formation, RNA extraction and qPCR analysis

To assess the potential for *in vitro* differentiation into cells of the three germ layers, mESCs lines were subjected to embryoid body (EB) formation following the protocol described by Ezekiel et al., 2007. First, feeder cells were removed from the mESCs cultures, as detailed above, and then dissociated single mESCs were resuspended in differentiation medium (KSR medium without R2i and LIF) and counted using a hemocytometer chamber. To achieve the formation of uniformly sized EBs, 1.000 cells in a 200 µL volume were dispensed into each well of sterile 96-conical well plates (ThermoFisher), filling 45 wells per mESCs line, tapped gently and incubated at 37°C and 5% CO_2_. Every 2 days, 100 µL of medium was removed and 100 µL of fresh differentiation medium was added to each well.

After 7 days, 20-30 EBs per mESCs line were collected to analyze the expression of pluripotency genes (*Oct4*, *Nanog,* and *Rex1*), endoderm lineage genes (*Afp, Foxa2, and Ttr*), mesoderm lineage genes (*T-Brachyury, Nkx2-5, and Kdr*) and ectoderm lineage genes (*Nes, Pax6,* and *Fgf5*) by quantitative PCR (qPCR).

Total RNA was extracted from trypsinized EBs using the Maxwell RSC SimplyRNA Tissue Kit according to the manufacturer’s instructions. RNA concentration and quality were assessed using a NanoDrop spectrophotometer. One microgram of the extracted RNA was reverse-transcribed to cDNA using the iScript cDNA Synthesis Kit (Bio-Rad).

The qPCR reactions were performed in triplicate on a CFX3846 Real-Time System thermocycler (Bio-Rad) using iTaq universal SYBR Green Supermix (Bio-Rad). The amplification program consisted of an initial denaturation step of 3 min at 95°C, followed by 40 cycles of 10 s at 95°C (denaturing) and 30 s at 60°C (annealing and extension). An additional thermal denaturing cycle was performed to obtain the melting curve of the qPCR products.

Validated commercial PrimePCR SYBR Green Assays (BioRad) for *Oct4* (Pou5f1, qMmuCED0046525), *Rex1* (*Zfp42*, qMmuCID0008767) and *Nanog* (qMmuCID0005399) were used to assess pluripotency. *Afp* (qMmuCID0023288), *Foxa2* (qMmuCED0048428) and *Ttr* (qMmuCID0006861), *T-Brachyury* (qMmuCID0015169), *Nkx2-5* (qMmuCID0020858) and *Kdr* (qMmuCID0005890), and *Nes* (qMmuCID0023067), *Pax6* (qMmuCID0026137), and *Fgf5* (qMmuCID0008576) were used to assess differentiation to endoderm, mesoderm and ectoderm lineages, respectively. A no-template control (NTC) was added for each primer and the results were normalized to *Gapdh* (qMmuCED0027497) expression. Additionally, the relative expression values in EBs samples were normalized to their respective undifferentiated ESC lines. The cycle quantification value (Cq-value) was determined for each sample using BioRad CFX Maestro™ 195 software, and relative expression was calculated using the ΔΔCq method.

### Statistical analysis

Aggregate formation rates were analyzed with Fisher’s exact test. The mean numbers of total, OCT4+ and NANOG + cells and the mean ratios of OCT4+/total cells, NANOG+/total cells and NANOG+/OCT4+ were first analyzed using the Shapiro-Wilk test to check the normality of the samples and then compared using either an unpaired *t*-test or a Mann-Whitney test. All the statistical analyses were performed using the GraphPad Prism 8 software and *p* < 0.05 was considered as statistically significant.

Mean values are indicated in the text, tables, and figures as mean ± SEM.

## Results

### Ability of sister blastomeres of 8-cell embryos to produce mESCs lines

**FIGURE 1:**
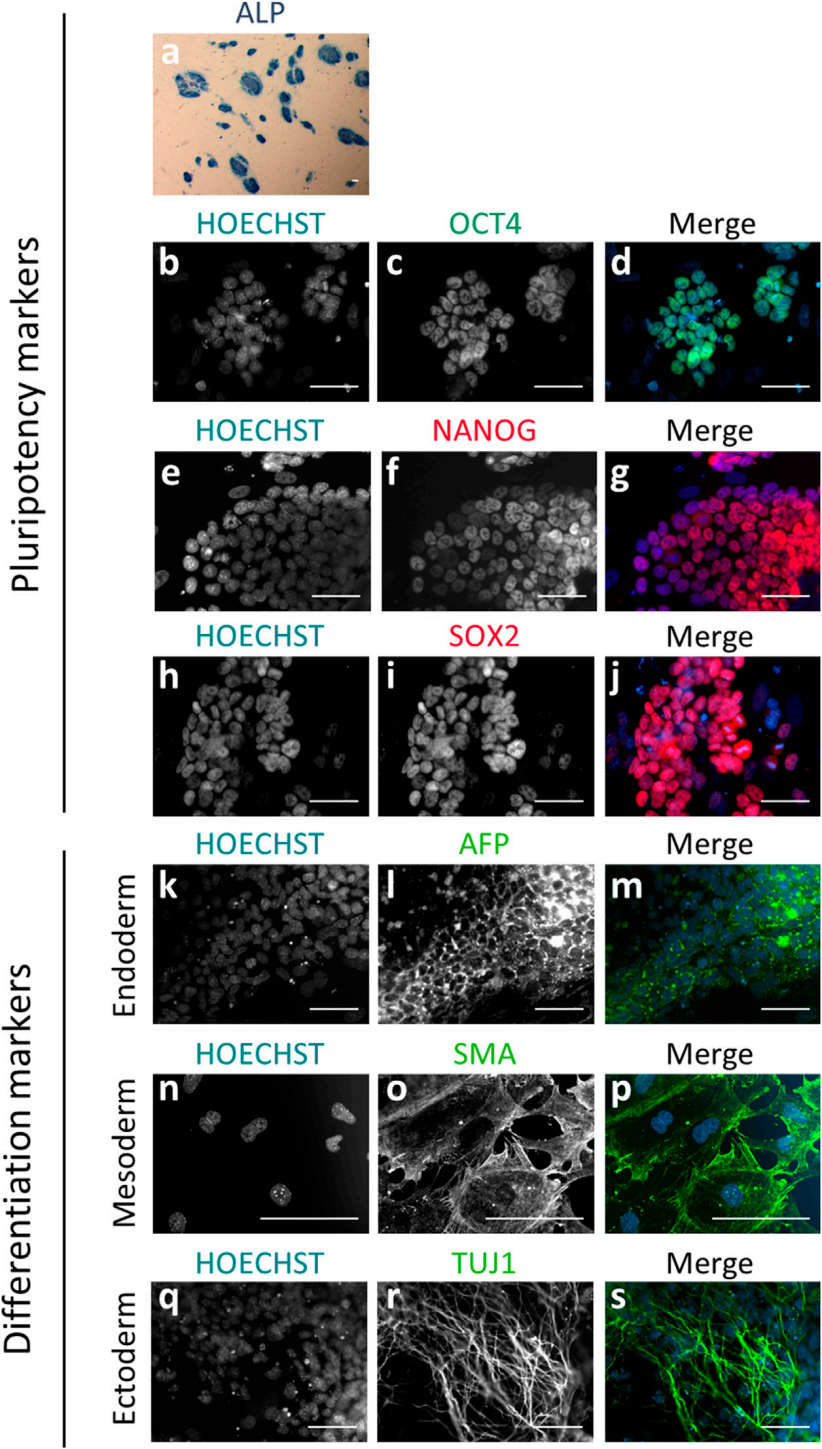
Immunodetection of pluripotency and differentiation markers on blastomere-derived mESC lines. **(A)** mESC colonies positive for alkaline phosphatase activity (ALP) showing blue staining. **(B–S)** Epifluorescence raw and merge images of mESC lines for pluripotency markers OCT4 **(B–D)**, NANOG **(E–G)** and SOX2 **(H–J)**, and differentiation markers for endoderm (AFP; k-m), mesoderm (SMA; n-p) and ectoderm (TUJ1; q-s) after spontaneous differentiation *in vitro*. In all the immunofluorescence images, nuclear material is counterstained with Hoechst (blue). All the scale bars represent 50 µm.

In the first set of experiments, we analyzed the ability of the individual blastomeres from the 8-cell embryo to yield mESCs. In total, 1.008 blastomeres from 126 embryos were biopsied in 13 replicate experiments, and individually cultured on a monolayer of iHFF cells in a medium containing KSR and the R2i inhibitor cocktail (KSR + R2i medium). We obtained a total of 292 mESCs lines (29%), whose stemness was confirmed at the seventh passage by morphological criteria (colonies with rounded morphology and defined edges), pluripotency criteria (positive for Alkaline Phosphatase (ALP) activity and OCT4, SOX2 and NANOG markers), and differentiation criteria (positive for TUJ1, SMA and AFP after *in vitro* spontaneous differentiation for 10 days) (Figure 1).

Of the total embryos, 84.1% (106/126) formed at least one mESCs line. Most of the embryos (97/126) gave rise to one to four lines, but two embryos were able to produce seven lines (Table 1). These results suggest that in the majority of embryos, 50% or less of blastomeres possess the potential to form a line of pluripotent mESCs under our derivation conditions, although in a few embryos this potential is expanded to almost all (87.5%) blastomeres.

**TABLE 1:** Number and percentage of 8-cell embryos producing different numbers of blastomere-derived mESCs lines.

Lines per embryo	Number of embryos (%)
0	20 (15.9)
1	22 (17.5)
2	26 (20.6)
3	27 (21.4)
4	22 (17.5)
5	7 (5.6)
6	0
7	2 (1.6)
8	0

### Ability of single blastomeres of 8-cell embryos to develop into cell aggregates with pluripotent potential

To assess whether the failure of some blastomeres within each embryo to produce mESCs lines was related to their lack of pluripotent potential, we next examined the ability of single blastomeres to develop into cell aggregates containing pluripotent EPI cells. Cell aggregates are the precursors of outgrowths, and because of their higher formation efficiency and earlier manifestation in the derivation process, their formation could be a better marker of blastomere developmental potential.

**FIGURE 2:**
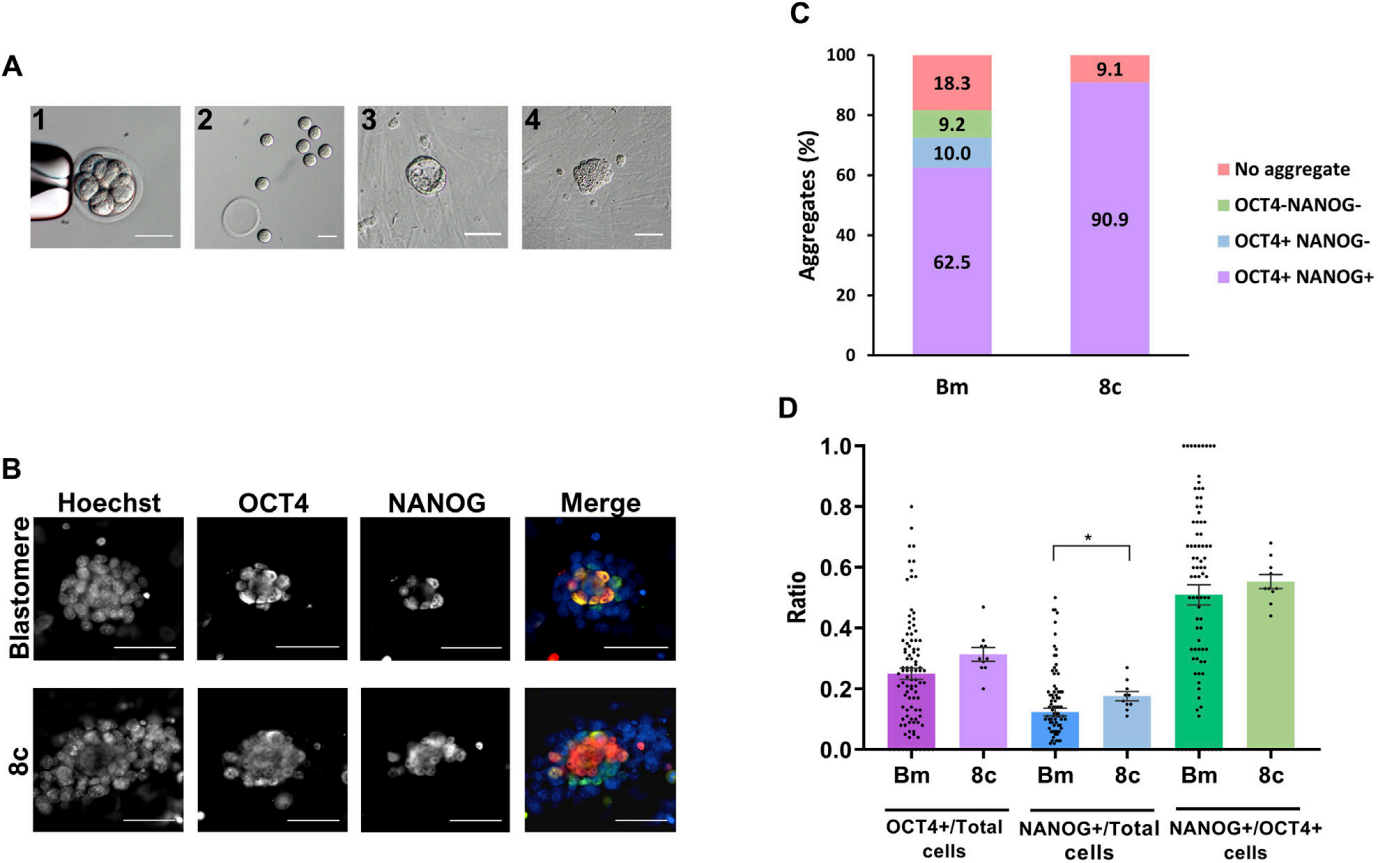
Pluripotency of cell aggregates derived from single blastomeres and control 8-cell embryos. **(A)** Eight-cell embryo before (1) and after (2) biopsy for the obtention of single blastomeres. After individual culture for 72 h, most blastomeres formed cell aggregates with (3) or without (4) cavity. All the scale bars represent 50 µm. **(B)** Epifluorescence raw and merge images of blastomeres-derived and 8-cell embryos-derived cell aggregates immunostained for OCT4 (green) and NANOG (red) and counterstained with Hoechst (blue). All the scale bars represent 30 µm. **(C)** Percentage of blastomeres (Bm) and control 8-cell embryos (8c) developed into cell aggregates positive or negative for NANOG and OCT4 markers. **(D)** Mean values (bar chart) ± SEM of the ratios of OCT4+/Total cells, NANOG+/Total cells and NANOG+/OCT4+ cells in aggregates formed from single blastomeres and control 8-cell embryos. Each black dot indicates the value of a single aggregate. Statistically significant differences were found between both groups in NANOG/Total cells ratios (*p* < 0,05, Mann-Whitney test).

In this second set of experiments, 120 blastomeres from 8-cell embryos (*n* = 15) were singly extracted by micromanipulation (Figures 2A.1, 2) in three replicate experiments, and individually cultured in KSR + R2i medium for 72 h. After 3 days in culture, 81.7% of the blastomeres (98/120) formed a cell aggregate, a percentage that did not statistically differ from the 90.9% of aggregates formed by control zona-free whole 8-cell embryos cultured under the same conditions (10/11). Some aggregates formed from single blastomeres (36.7%, 36/98) displayed a cavity (Figure 2A.3), similar to that of blastocysts, but the majority did not cavitate (63.3%, 62/98) (Figure 2A.4). These percentages were similar in aggregates formed from the control 8-cell embryos: 30% (3/10) with cavity and 70% (7/10) without cavity (Supplementary Figure S1). To investigate whether aggregate cavitation was related to developmental potential, we next evaluated the presence of ICM and EPI cells in the two types of cell aggregates. To this end, we immunostained all aggregates for the detection of OCT4 and NANOG markers. The percentage of aggregates that contained at least one OCT4-positive (OCT4+) cell or one NANOG-positive (NANOG+) cell (Supplementary Table S1), the mean number of total, OCT4+, and NANOG + cells (Supplementary Table S2), and the mean ratios of OCT4+/Total cells, NANOG+/Total cells and OCT4+/NANOG + cells (Supplementary Table S3) did not significantly differ between cavitated and non-cavitated aggregates. A similar result was observed when comparing cavitated and non-cavitated aggregates formed from control 8-cell embryos (Supplementary Tables S1–S3). According to these results, no differences in the developmental potential of cell aggregates with or without cavity seem to exist, thus, from this point on, the two types of aggregates were grouped.

In control 8-cell embryos, all aggregates formed contained both OCT4+ and NANOG + cells, indicating that 90.9% of the embryos (10/11) were able to develop into cell aggregates containing pluripotent EPI cells after 72 h of culture. This ability was reduced in single blastomeres, as only 62.5% of the initial blastomeres (75/120) developed into aggregates containing OCT4+ and NANOG + cells, even though 72.5% (87/120) of the blastomeres formed aggregates with OCT4+ cells (Figures 2B, C). It must be noted that, in both the control and blastomeres groups, all aggregates that were positive for NANOG were also positive for OCT4.

When comparing the mean ratios of OCT4+/Total cells, NANOG+/Total cells and NANOG+/OCT4+ cells between the aggregates derived from single blastomeres (0.25 ± 0.02, 0.12 ± 0.01 and 0.51 ± 0.03, respectively) and those derived from control 8-cell embryos (0.31 ± 0.02, 0.18 ± 0.02 and 0.55 ± 0.02, respectively), statistically significantly differences were found only in the NANOG+/Total cells ratio (Figure 2D).

Altogether, these results suggest that single blastomeres isolated from 8-cell embryos have the same potential as whole 8-cell embryos to develop into cell aggregates, but the specification of pluripotent EPI is slightly impaired.

### Distribution of the ability to develop into cell aggregates with pluripotent potential among sister blastomeres of 8-cell embryos

Next, we sought to determine how the potential to develop into cell aggregates containing pluripotent cells was distributed among the different blastomeres of the 8-cell embryo. For this purpose, the results obtained from single blastomeres were grouped according to their parental embryos.

**FIGURE 3:**
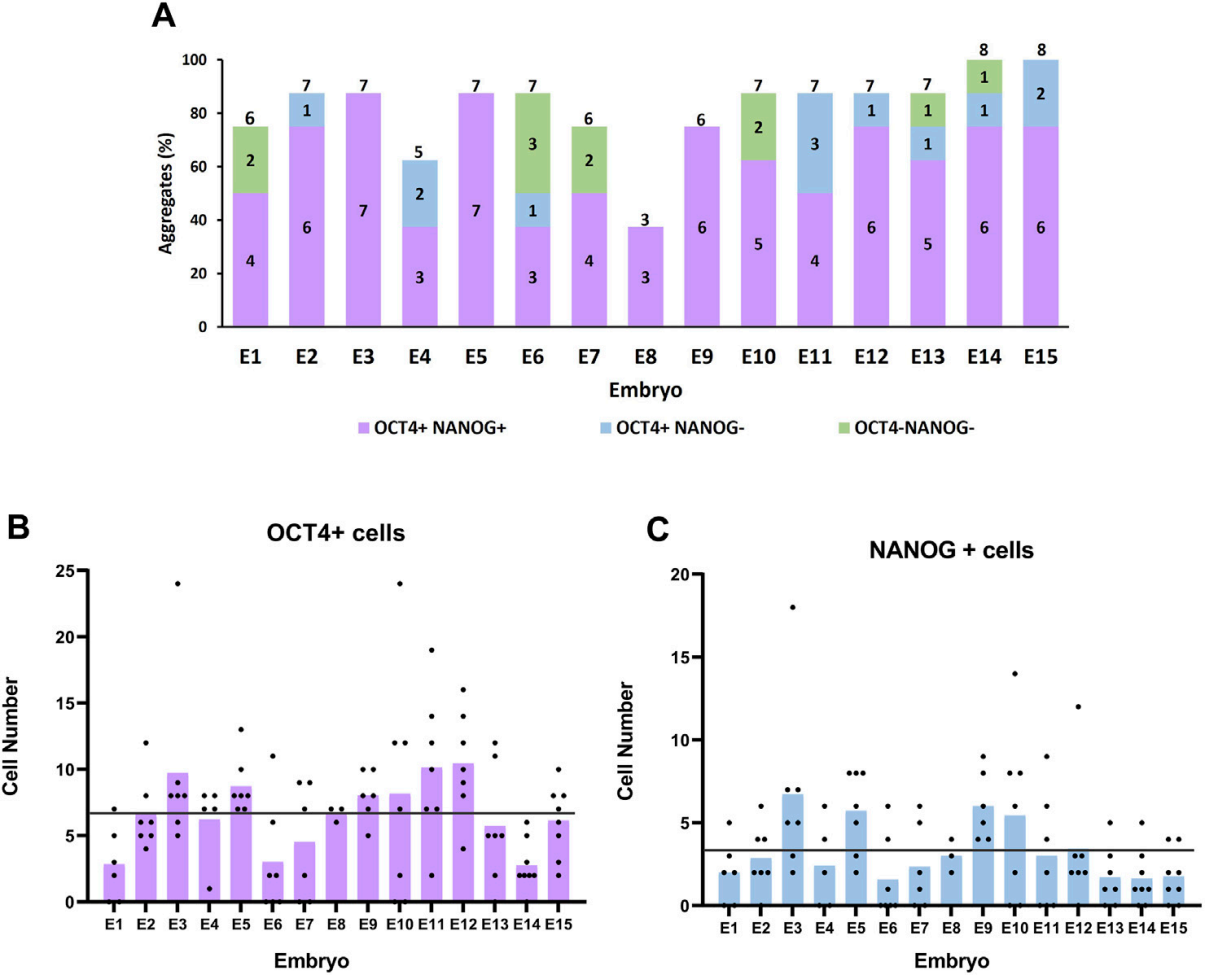
Formation and pluripotency of cell aggregates derived from sister blastomeres of 8-cell embryos. **(A)** Percentage (bar height) and number (numbers inside the bars) of cell aggregates positive or negative for OCT4 and NANOG markers formed by the sister blastomeres of each parental embryo. Numbers on top of the bars indicate the total number of aggregates formed per parental embryo. **(B, C)** Number of OCT4+ and NANOG + cells in cell aggregates formed from sister blastomeres of each parental embryo. Each black dot indicates the cell number for a single aggregate and each bar represents the mean cell number in each parental embryo. The horizontal lines indicate the mean cell number in all the parental embryos.

The number of single blastomeres per embryo that developed into a cell aggregate after 72 h in culture ranged from 3 to 8, with a mean number of 6.5 ± 0.3 (Figure 3A). Most embryos (11/15) produced six to seven aggregates, and two were able to form eight aggregates. These results indicate that most, if not all, blastomeres of the 8-cell embryo have the potential to develop and form a cell aggregate under our culture conditions.

The sum of aggregates formed by the sister blastomeres of the same parental embryo had an average total number of 190.5 ± 16.8 cells (222.8 ± 15.9 if only embryos forming seven to eight aggregates are considered), a value significantly higher than the average total number of cells in control 8-cell embryo-derived aggregates (95.2 ± 10). These results indicate that when blastomeres from the same embryo are isolated and cultured separately, they can generate a higher number of cells than when they remain together.

The mean number of aggregates per embryo that were positive for both OCT4 and NANOG was 5.0 ± 0.4, ranging from 3 to 7 (Figure 3A). Thus, the percentage of blastomeres within an embryo that possesses the capacity to form aggregates containing NANOG + cells varied largely among embryos, from 37.5% (three NANOG + aggregates) to 87.5% (seven NANOG + aggregates). However, most commonly (7/15), embryos formed six to seven cell aggregates containing NANOG + cells, meaning that 75.0%–87.5% of the blastomeres of these embryos had the ability to form aggregates with pluripotent EPI cells.

The number of OCT4+ and NANOG + cells in the cell aggregates was heterogeneous and showed large variation both among sister blastomeres and among parental embryos (Figures 3B, C). The number of ICM cells (OCT4+) varied from 0 to 24 in the aggregates produced from the different blastomeres, with a mean of 6.7 ± 0.5, and the number of EPI cells (NANOG+) varied from 0 to 18, with a mean of 3.3 ± 0.3. These values were significantly lower than the mean number of OCT4+ cells (29.5 ± 3.3; range 15–50) and NANOG + cells (16.3 ± 2.1; range 8–32) present in the cell aggregates formed by control whole 8-cell embryos. Interestingly, however, when all the aggregates formed by the sister blastomeres of each parental embryo were grouped, the mean numbers of OCT4+ and of NANOG + cells in the sum of aggregates (43.5 ± 5.1 and 21.5 ± 3.2, respectively) were higher but did not significantly differ from the mean numbers in control whole 8-cell embryos-derived aggregates (29.5 ± 3.3 and 16.3 ± 2.0, respectively).

Finally, given the variation in the number of pluripotent EPI cells among sister blastomeres and that only 29% of the blastomeres were able to produce an mESCs line in the first set of experiments, we tried to estimate the minimum number of NANOG + cells required for the successful establishment of an mESCs line from single blastomeres. To do this, we classified the cell aggregates formed by all the individual blastomeres according to the number of NANOG + cells they contained and selected the 29% upper range, assuming that mESCs lines would most probably be formed from cell aggregates possessing a higher number of NANOG + cells. This 29% upper range contained 5–18 NANOG + cells, indicating that single blastomeres should generate cell aggregates containing at least five NANOG + cells after 3 days in culture for them to be able to subsequently establish an mESCs line. Considering that in the first set of experiments the majority of the embryos (97/126; 77.0%) generated one to four mESCs lines, we next calculated the percentage of embryos in the second set of experiments that were able to form one to four aggregates containing the estimated minimal number of NANOG + cells (≥5). Interestingly, the percentage was very similar (73.3%; 11/15).

### Transcriptomic variations among mESCs lines derived from sister blastomeres of 8-cell embryos

**FIGURE 4:**
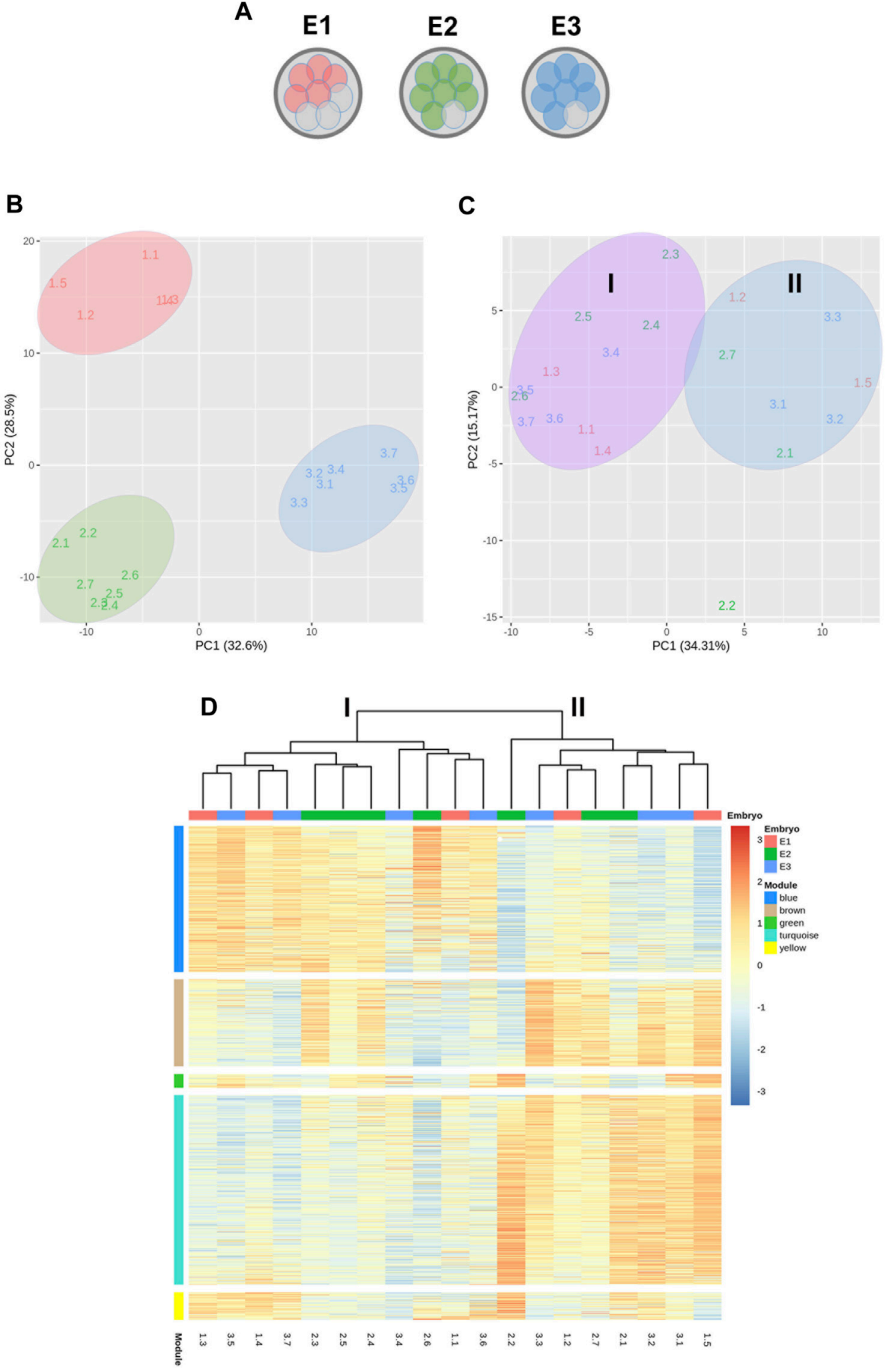
Transcriptomic analysis by RNA-seq of 19 mESCs lines derived from single blastomeres. **(A)** Diagram representing the mESCs lines used in RNA-seq analysis, obtained from three different embryos. **(B)** Principal component analysis (PCA) showing the clustering of the mESCs lines according to their parental embryo. **(C)** PCA after between-embryo variation correction, showing lines segregating in two clusters, labeled as group I and II. **(D)** Heatmap of weighted gene correlation network analysis (WGCNA) of genes with variance >0.05. Five modules of highly correlated genes were identified: blue, brown, green, turquoise and yellow.

To characterize and compare the molecular features of mESCs lines produced from different blastomeres of the same parental embryo in terms of their gene expression patterns, we selected the lines produced by embryos that had given rise to the largest number of mESCs lines in the first set of experiments. In particular, five mESCs lines established from one embryo and seven mESCs lines established from two other embryos (total mESCs lines = 19) were selected and subjected to RNA sequencing (RNA-seq) (Figure 4A). Two interesting observations emerged from the analysis of their transcriptomes. First, PCA revealed that the lines clearly clustered into three separate groups according to the parental embryo from which the blastomeres originating these lines were biopsied (Figure 4B). Second, although the majority of genes expressed in embryos were protein coding (Supplementary Figure S2), a heatmap of the top 50 most representative differentially expressed genes (Supplementary Figure S3) revealed a high frequency of differential pseudogenes expression among the three embryos. Most of these pseudogenes were related to ribosomal protein genes.

To reveal possible transcriptional variations among blastomeres-derived mESCs other than those related with the parental embryo of the blastomeres, between-embryo variability was corrected and a new PCA was performed. This new analysis revealed that lines segregated into two clusters (Groups I and II) and, interestingly, half of the lines from each embryo were allocated to each cluster (Figure 4C), except for one line derived from embryo 2 (line 2.2).

**FIGURE 5:**
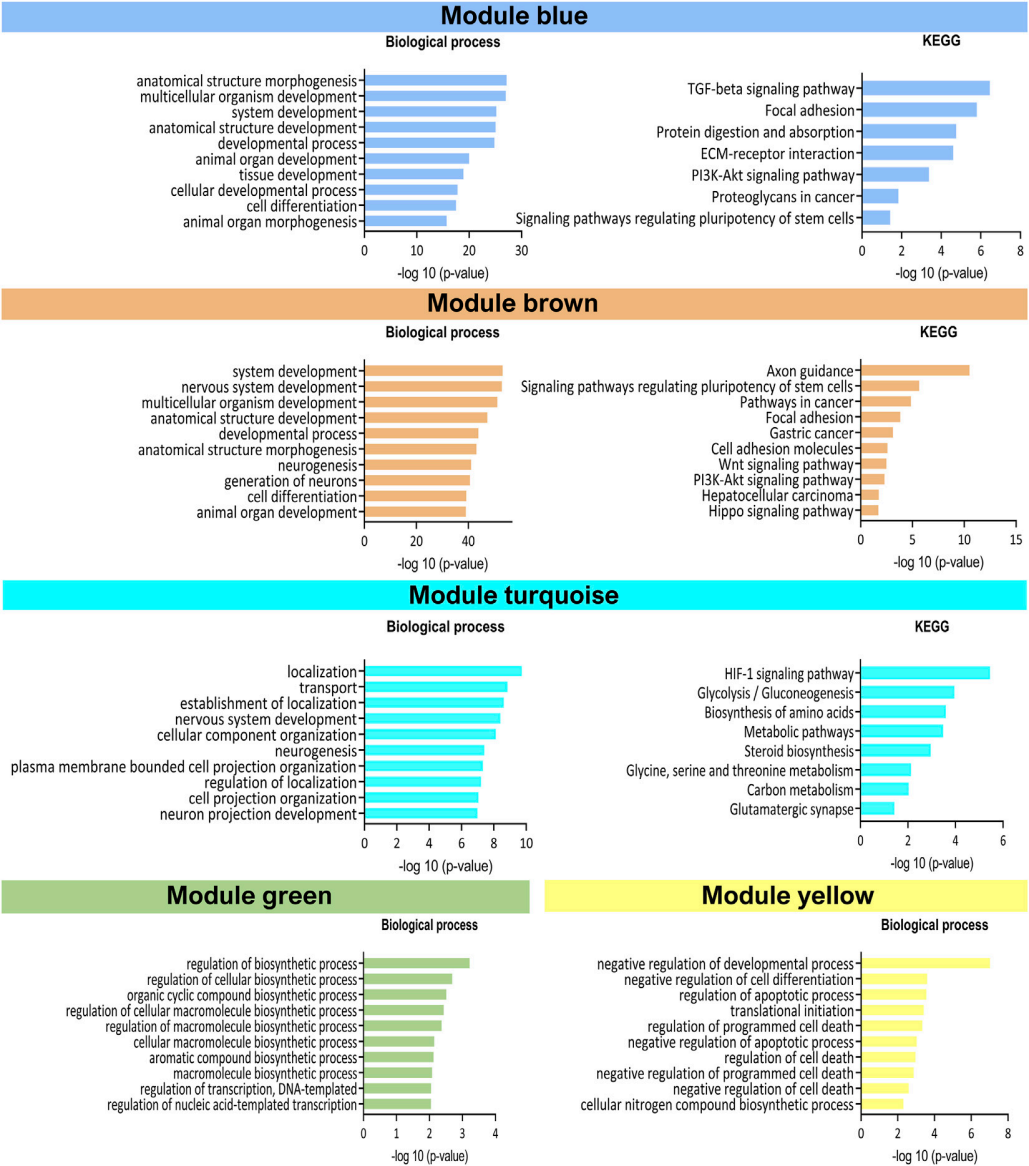
Representation of Gene Ontology enrichment analysis in the five WGCNA modules. Top 10 significantly enriched (-log10 (*p*-value)) GO process in GO biological process terms and KEGG database.

Next, a WGCNA was performed on the embryo-adjusted gene counts, in which mESCs line 2.2 was shown to cluster together with lines in group II. Using this analysis, we identified five clusters of highly correlated genes (blue, brown, green, turquoise, and yellow modules) (Figure 4D), which are involved in different processes and pathways (Figure 5). In module blue (1,104 genes), upregulated in group I, GO enrichment analysis revealed differential expression of genes involved in developmental and morphogenesis process, such as anatomical structure, tissue, organ, and cell development, regarding GO term biological process. Analysis of KEGG (Kyoto Encyclopedia of Genes and Genomes) and WikiPathways (WP) databases, revealed upregulation of signaling pathways associated with stem cell pluripotency, such as TGF-β and PI3k-Akt. By contrast, lines from Group II displayed upregulation of genes from the turquoise module (1,431 genes), related with transport and localization process, as well as in nervous system development, neurogenesis, and neuron projection development, in terms of GO biological process. Regarding the KEGG or WP databases, biosynthesis of amino acids and metabolic pathways, such as glycolysis and gluconeogenesis were processes upregulated in this module. Whereas transcriptional differences in genes included in modules blue and turquoise were evident between mESCs lines belonging to group I and those belonging to group II, differences were not as evident for genes in modules brown, green, and yellow, with some lines in each group showing upregulated and others showing downregulated expression of genes included in each of these three modules. Module brown (658 genes) was enriched in genes that play a role in biological processes such as development process as well as in neuron system development and generation of neurons, also consistent with the analysis performed with the KEGG database. Genes in module green (105 genes) were related with biosynthetic process and, finally, module yellow (208 genes) was enriched in transcripts involved in apoptotic process and cell death. Altogether, the results of these analyses indicate that mESCs lines derived from different blastomeres of the 8-cell embryo can be split into two different groups according to their transcriptional profiles, with one group showing upregulated expression of genes related with pluripotency and development.

**FIGURE 6:**
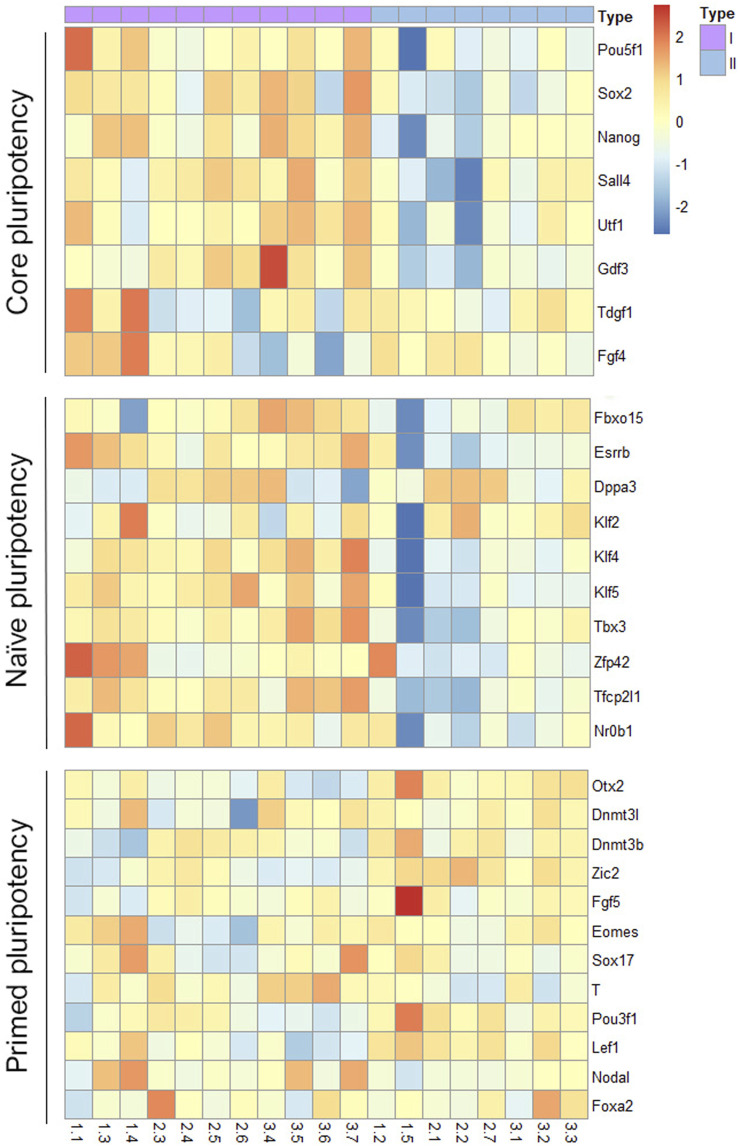
Heatmap representation of pluripotency gene expression in the two groups of mESCs lines. Differential expression of core, naïve and primed pluripotency markers in the two groups of blastomere-derived mESCs lines identified after between-embryo effect correction.

Finally, to visualize how the two groups of mESCs lines differed in their pluripotency potential, we produced a heatmap of the differential expression of several core, naïve and primed pluripotency genes (Figure 6). Lines in group I showed upregulated expression of core and naïve pluripotency genes and downregulated expression of primed pluripotency genes compared with lines from group II. These results suggest that among the mESCs lines generated from sister blastomeres of the 8-cel embryo, half possess a higher pluripotency potential than the other half, according to their transcriptional profiles.

### Formation of cells of the three primary germ layers from mESCs lines obtained from sister blastomeres of 8-cell embryos

**FIGURE 7:**
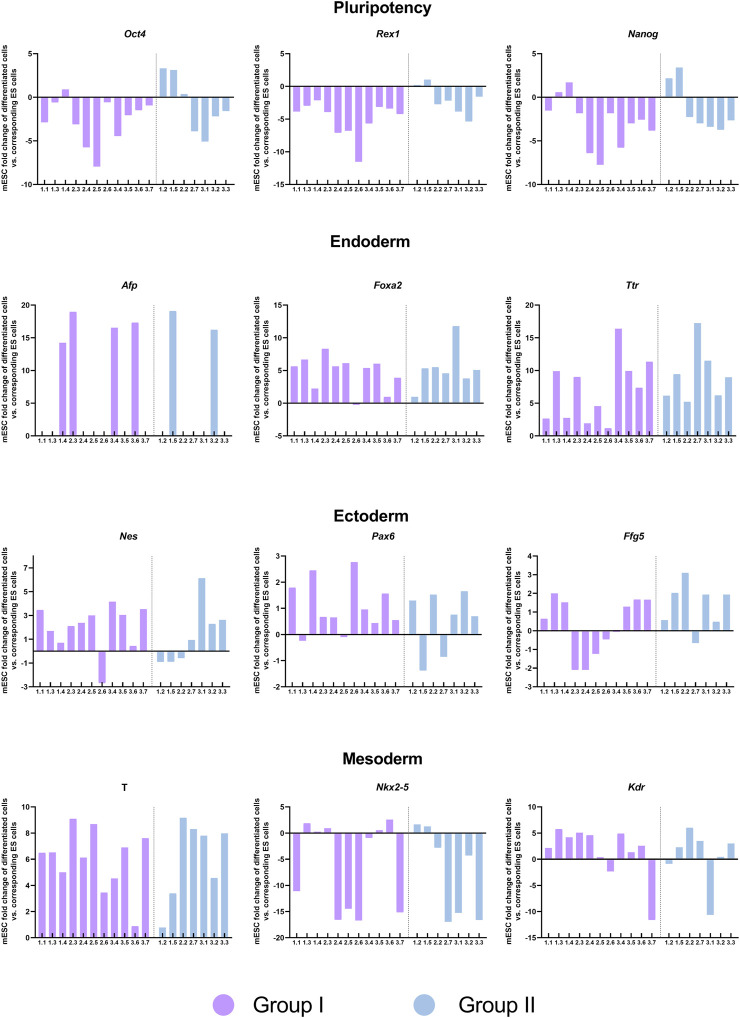
Expression of pluripotency and germ layer-specific genes in embryoid bodies generated from the blastomere-derived mESCs lines. Expression of pluripotency, endoderm, ectoderm and mesoderm markers in EBs analyzed by qPCR and normalized to their corresponding undifferentiated lines. The two groups of mESCs lines identified in the transcriptomic analysis are shown in different colors.

Finally, we sought to determine whether the transcriptional differences observed between the mESCs lines generated from single blastomeres (groups I and II) would create a bias in their ability to form cells of the three developmental germ layers. To this end, the same blastomere-derived mESCs lines used in the RNA-seq analyses were subjected to spontaneous *in vitro* differentiation for EB formation, and the expression of pluripotency (*Oct4*, *Nanog,* and *Rex1*), endoderm lineage (*Afp, Foxa2, and Ttr*), mesoderm lineage (*T-Brachyury, Nkx2-5,* and *Kdr*) and ectoderm lineage (*Nes, Pax6,* and *Fgf5*) marker genes was measured by qPCR on the EBs formed. All but one of the mESCs lines (line 2.1, a member of group II) were able to form EBs. Heterogeneity in the expression of these markers was evident among mESCs lines, regardless of the group they belonged to or the parental embryo they originated from (Figure 7). Although mESCs lines from group I exhibited a higher reduction in pluripotency gene expression (*Oct4, Rex1, Nanog*) upon differentiation, when compared with mESCs lines from group II, no biased differentiation towards a specific lineage was found among the mESCs lines of the two groups.

## Discussion

The derivation of ESCs lines from single blastomeres instead of whole blastocysts offers some potential advantages, such as the possibility of preserving the viability of the parental embryo, obtaining multiple lines from a single embryo, or producing lines with higher developmental and differentiation potential. In the first two cases, blastomeres of 8-cell embryos would be a better choice than blastomeres from earlier embryonic stages. Nonetheless, it is not known whether all blastomeres of an 8-cell embryo are equally capable of producing an ESCs line and this is a relevant question in terms of derivation efficiencies and potential efforts to improve them. Although several groups have succeeded in deriving ESC from 8-cell human and mouse embryos (Klimanskaya et al., 2006; Chung et al., 2008; Taei et al., 2013; Hassani et al., 2014b; Yang et al., 2017; Vila-Cejudo et al., 2019; Alonso-Alonso et al., 2022), the low number of embryos used or randomization of blastomeres obtained from the different embryos have precluded answering this question. Moreover, although the developmental potential of single blastomeres of 2-cell and 4-cell stage embryos has been explored in several studies, blastomere potency at the 8-cell stage has seldom been investigated (Boiani et al., 2019). Here, we attempted to answer this question by employing a high number of embryos (*n* = 126) and blastomeres (*n* = 1.008) and by keeping track of the parental embryo of each blastomere. We first analyzed the potential of sister blastomeres of 8-cell embryos to produce mESCs lines and cell aggregates containing pluripotent cells (as precursors of mESCs lines). To maximize the development of blastomeres into cell aggregates, outgrowths and mESCs lines, isolated blastomeres were cultured on feeder cells and in the presence of LIF and R2i in the culture medium (Alonso-Alonso et al., 2022).

The percentage of embryos that were able to produce at least one mESCs line (84.1%) was similar to the 85.3% derivation rate obtained by our group from whole blastocysts of the same mouse strain (Vila-Cejudo et al., 2019). Nonetheless, because most of the parental 8-cell embryos yielded more than one mESCs line, the total number of lines derived from the isolated blastomeres (292) was almost three times the number of lines we would have produced had we used the intact embryos (107 lines from 126 embryos, according to an 85% derivation rate). We were unable to obtain eight mESCs lines from a single embryo, but two embryos generated seven mESCs lines. Remarkably, to date, this is the highest number of mESCs produced from a single embryo reported in the literature.

The large variation in the number of mESCs lines obtained per embryo (0-7) suggests that blastomeres from 8-cell stage embryos are not equally competent at establishing mESCs lines and that the number of competent blastomeres is not uniformly distributed among embryos. Nonetheless, among the embryos that produced at least one mESCs line, those in which the mESCs generation potential was restricted to half or less of the blastomeres were much more abundant (91.5%) than those in which more than 50% of the blastomeres produced an mESCs line (8.5%). To the best of our knowledge, no similar studies have been performed with 8-cell embryos, but Lorthongpanich et al. (2008) also found that in the majority of 4-cell stage embryos only 50% or less of the blastomeres were able to produce outgrowths with visible ICM clumps. Similarly, Casser et al. (2017) demonstrated that blastomeres in most 2-cell mouse embryos displayed unbalanced abilities to generate mESCs lines, although this ability was concordant between the two blastomeres in approximately 30% of the embryos.

One of the limiting factors during mESCs establishment from whole blastocysts is the rapid loss of pluripotent EPI cells, the progenitors of mESCs, during the first few days of culture (Buehr and Smith, 2003). Therefore, we next examined the development of blastomeres at an earlier point in the derivation process (day 3 of culture), to determine their ability to produce pluripotent EPI cells before this culture-induced loss occurred. We again found large variability among embryos in the number of blastomeres that developed into OCT4+ and NANOG + cell aggregates (3-7). However, in this case, embryos in which more than half of the blastomeres were capable of developing into cell aggregates containing EPI cells were more abundant (60%) than those in which half or less of the blastomeres showed this capacity (40%). Moreover, a large variation in the number of EPI cells per aggregate was found among blastomeres, and the percentage of embryos that produced at least one NANOG + cell aggregate (100%) was higher than that of embryos that produced at least one mESCs line in the first set of experiments (84.1%). Altogether, these results led us to assume that the limiting factor for a blastomere to be able to produce an mESCs line should reside in its ability to produce a minimum number of pluripotent EPI cells rather than in its ability to simply generate a NANOG + cell aggregate, and we estimated this minimum number to be 5 cells. Lineage-tracing experiments should be performed in future studies to confirm it. However, interestingly, this number is similar to four or more EPI cells that have been reported to be required by the time of implantation for successful development to term (Morris et al., 2012).

Several studies have shown that blastomeres isolated from most precompaction embryos differ in their ability to form TE, EPI, and PE cells, indicating their unequal developmental potential (Krawczyk et al., 2021; Maemura et al., 2021). A developmental bias among sister blastomeres has also been reported in intact 4-cell and 8-cell mouse embryos, in which lineage tracing experiments demonstrated that most individual embryos contain blastomeres exhibiting biased and unbiased contribution patterns to TE and ICM and their derivatives (Tabansky et al., 2013). Other lineage tracing experiments in intact 2-cell and 4-cell embryos have also revealed that, on average, half of the cells of the embryo preferentially contribute to TE, while the other half contribute preferentially to ICM (Jedrusik et al., 2008). Considering these results, it would seem reasonable to assume that blastomeres that gave rise to mESCs lines in our study were those with an intrinsically higher developmental potential in the intact embryo. In this scenario, a biological constraint could be limiting the generation of mESCs to only half or less of the blastomeres of most 8-cell embryos, although this number could be further reduced by culture conditions that do not allow to maintain the inherent developmental potential of some blastomeres during the mESCs derivation process. Indeed, the maximum rate of mESCs derivation achieved so far from the blastomeres of 8-cell embryos is 50% (Hassani et al., 2014a). However, because the culture conditions used in this study were very similar to ours, these results could probably be attributed to the much lower number of blastomeres used (*n* = 72) compared with our study (*n* = 1.008), rather than to the use of ideal experimental conditions for the maintenance of blastomere developmental potential.

In addition to this biological constraint, the plasticity of embryonic development and the presence of signaling modulators in the derivation medium should also be considered when interpreting the results of our study. Blastomeres of 8-cell embryos show impaired ability to form pluripotent EPI cells after isolation and culture in standard embryo culture medium, and they tend to develop as trophoblastic vesicles instead of forming blastocysts (Krawczyk et al., 2021; Maemura et al., 2021). This could be attributed to the lack of cell-cell contacts and positional information, which was shown to induce blastomeres to change their gene expression pattern towards a unique pattern characteristic of neither ICM nor TE, but with a tendency to mimic that of TE cells (Lorthongpanich et al., 2012). This could explain the extremely low rates of mESCs derivation from isolated blastomeres of 8-cell embryos (Wakayama et al., 2007; González et al., 2010) and their partial rescue when incubating the blastomeres for 24 h with E-cadherin to mimic cell-cell contacts (González et al., 2011b). Similarly, the addition of 2i or R2i to the culture medium significantly improves mESCs derivation rates from single 8-cell blastomeres (Hassani et al., 2014b; Vila-Cejudo et al., 2020; Alonso-Alonso et al., 2022). The signaling modulators present in these cocktails have been shown to expand the pluripotent EPI population in intact embryos and isolated blastomeres (Nichols et al., 2009; Van Der Jeught et al., 2014; Alonso-Alonso et al., 2022) and their effects on the maintenance of ESCs pluripotency are well known (Ying et al., 2008). Moreover, MEK inhibition has been shown to promote the retention of pluripotent cells during the outgrowth phase of mESCs derivation (Buehr and Smith, 2003). Therefore, the use of R2i in our mESCs derivation medium could be promoting the maintenance of the pluripotent potential or the reversal of their pre-determined non-EPI fate after isolation and the loss of cell-cell contacts. Considering all this, the mESCs derivation rates obtained in the present study are probably the result of a combination of the biological constraints in terms of developmental potential among sister blastomeres, the developmental plasticity of the blastomeres and the culture conditions. Efforts to increase derivation rates should therefore be focused on improving experimental protocols to maintain pluripotency after isolation in blastomeres with higher developmental potential in the intact embryo and to promote the redirection of cell fate towards the EPI lineage in those ones with lower developmental potential. Alternatively, groups of 2 or 3 blastomeres from each 8-cell embryo instead of a single one could be used to ensure the generation of the minimum number of EPI cells required for the successful establishment of a mESCs line, as demonstrated by Morris et al (2012).

Next, we were interested in determining whether mESCs lines produced from different blastomeres of the same embryo are equivalent in terms of their transcriptional signature. To the best of our knowledge, only one study has addressed this question, using mESCs derived from single blastomeres of 8-cell human embryos (Zdravkovic et al., 2015). We found that the transcriptional profile of the mESCs lines differed according to the parental embryo from which they were established, in contrast to the findings of Zdravkovic et al (2015). This discrepancy may be attributable to genetic differences among embryos, as all human embryos used in the previous study were collected from a single couple whereas our embryos were collected from different donor hybrid F1 mice. F1 animals are genetically identical (heterozygous at all loci where the inbred parental strains differ) but F2 embryos are genetically distinct as a result of meiotic recombination and random chromosome segregation during meiosis, and may bear different contributions from the two parental inbred strains. Surprisingly, we also found a high number of pseudogenes in the top 50 differentially expressed genes among the mESCs derived from different embryos. Although pseudogenes cannot produce functional proteins, it has been reported that some can be transcribed and may play a role in regulating the expression of protein-coding genes (Pink et al., 2011). Most of them are processed pseudogenes, generated by highly expressed housekeeping genes (Pink et al., 2011); therefore, it is not surprising that most of the pseudogenes identified in our analyses were related to genes encoding ribosomal proteins. However, the fact that so many pseudogenes are present among the top differentially expressed genes in mESCs lines derived from the different embryos deserves further investigation, which is beyond the scope of the present study.

When we removed between-embryo variability, differences at the transcriptional level between mESCs lines derived from the same embryos became apparent, and interestingly, the lines consistently segregated into two distinct groups. When compared with the lines in group II, the lines in group I displayed upregulation of genes involved in developmental and morphogenesis processes and in signaling pathways associated with stem cell pluripotency, and of core and naïve pluripotency genes. This suggests that half of the mESCs lines generated from each embryo possess a higher pluripotency potential than the other half. Because the derivation of mESCs lines is a long process, it is not possible to completely rule out that differences in the lines’ transcriptomes were acquired during the generation of the line and extended culture. However, the fact that the same pattern was observed in all three parental embryos and that a high number of lines were analyzed for each embryo (5-7), rather suggests that these differences stem from the blastomere that the lines originated from and were maintained during derivation and culture. It is tempting to speculate that lines in group I could have been established from the blastomeres that in the intact embryo possessed the highest developmental potential, whereas lines in group II could derive from those blastomeres with lower developmental potential in which redirection of cell fate towards the EPI lineage after isolation and culture successfully occurred. Because most of the lines used in the transcriptome analysis were obtained from two embryos in which 7 of the 8 blastomeres produced mESCs lines, this reinforces the idea that in the intact 8-cell stage embryo about half of the blastomeres would belong to each of these two categories. It would be interesting to investigate transcriptional differences among sister blastomeres of the 8-cell embryo in future studies to corroborate this idea. Zdravkovic et al. (2015) also found differences in gene expression among lines derived from the same embryo and related them to heterogeneities among the parental sister blastomeres, but the results of the two studies are difficult to compare in terms of the particular genes differently expressed because of the different species of the mESCs lines and the primed pluripotency of hESCs *versus* the naïve pluripotency of mESCs lines. Moreover, only two of the four 8-cell embryos used in this previous study had produced more than two lines, and the other two embryos had resulted in only one line.

Finally, despite the higher pluripotency of mESCs lines belonging to group I and the upregulated expression of genes related to nervous system development in lines belonging to group II, the two groups of lines did not show biased differentiation into any of the three primary germ layers. Terminal differentiation into derivatives of the three germ layers could be performed in future experiments for a more definitive assessment of whether a correlation exists between the transcriptional differences between the two groups of lines and their differentiation potential.

In summary, we have demonstrated that it is possible to obtain multiple mESCs lines from the blastomeres of a single 8-cell embryo, but that blastomeres at this developmental stage are unequally competent at producing mESCs lines. We estimated that this competence is related to their ability to produce at least five EPI cells during the first 3 days of culture. Our results also suggest that the current derivation rates probably reflect a combination of three factors: biological constraints, developmental plasticity, and culture conditions. Although in most embryos up to half of the blastomeres would have a higher developmental potential (thus, more competent at mESCs generation), isolation and culture of the blastomeres could reduce this potential. This could be prevented by culture conditions, such as the use of R2i, which could also allow to “rescue” those blastomeres with reduced developmental potential, by redirecting their cell fate to the EPI lineage. It is possible that the two distinct types of transcriptional signatures identified in the mESCs lines produced from the same embryos reflect the intrinsic developmental potential of the parental blastomeres from which they originated. However, these two different transcriptional signatures did not affect the ability of the lines to differentiate into cells of the primary germ layers. Our findings may help improve the generation of mESCs from single blastomeres and the understanding of cell fate determination in the preimplantation mouse embryo.

## Data Availability

The datasets presented in this study can be found in online repositories. The names of the repository/repositories and accession number(s) can be found below: https://www.ncbi.nlm.nih.gov/geo/, GSE233762.
