# Supplementary material for: Blastomeres of 8-cell mouse embryos differ in their ability to generate embryonic stem cells and produce lines with different transcriptional signatures
Source: Front Cell Dev Biol. 2023 Oct 9;11:1274660. doi: 10.3389/fcell.2023.1274660 (PMC10591181; doi:10.3389/fcell.2023.1274660)
Supplement: Supplementary file 1 [file Datasheet1.PDF]

Supplementary information

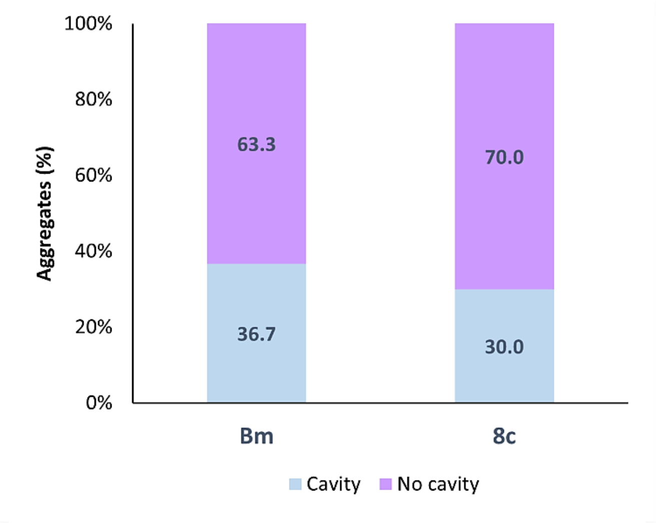

Fig. S1. Cavitation in cell aggregates. Percentage of cell aggregates with and without cavity obtained from single blastomeres (Bm) and from control zona-free 8-cell embryos (8c).

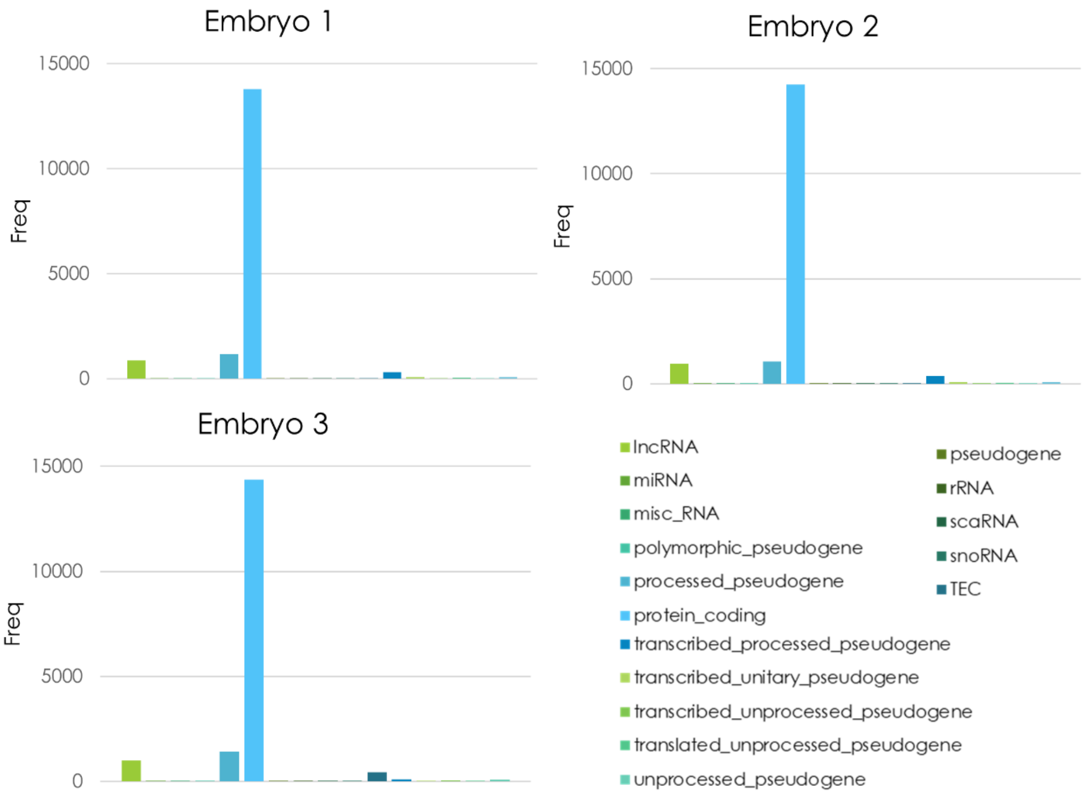

Fig S2. Types of genes expressed by mESCs lines derived from single blastomeres of three different embryos. Frequency of different types of genes expressed by all the mESCs lines generated from each of the embryos analyzed.

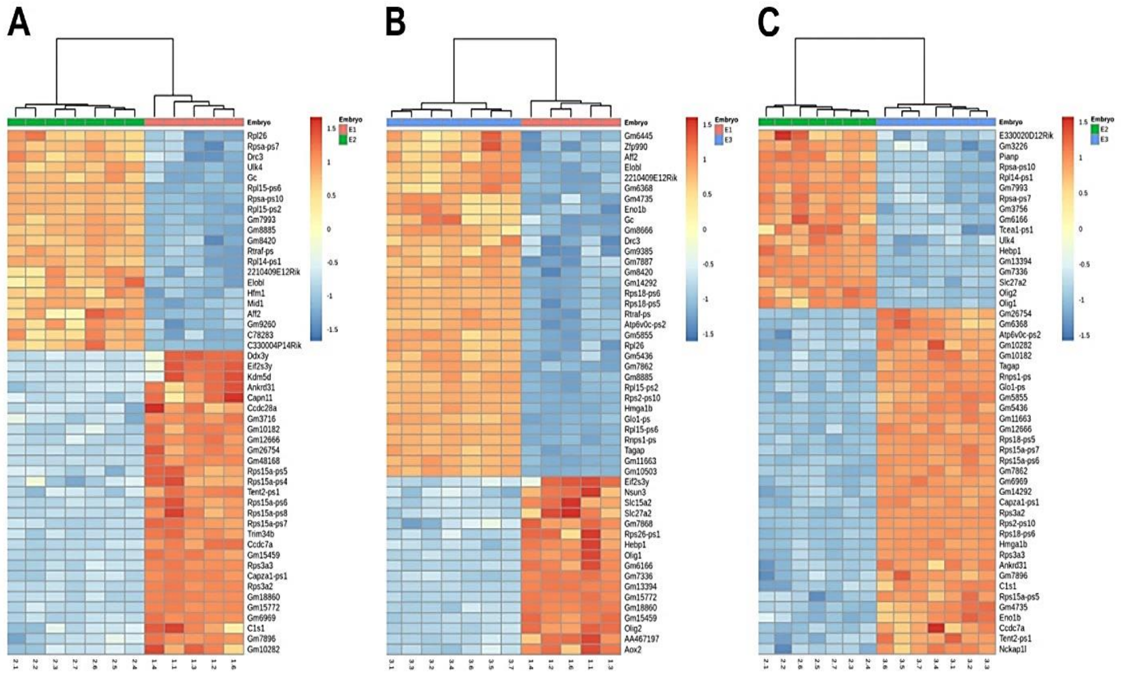

Fig. S3. Heatmap of the top 50 differentially expressed genes among mESCs lines derived from single blastomeres of three different embryos. In each heatmap, mESCs lines derived from the blastomeres of one embryo are compared with mESCs lines derived from the blastomeres of another embryo (A: E1 vs. E2; B: E1 vs. E3; C: E2 vs. E3). For each comparison, the name of the top 50 differentially expressed genes is indicated on the right.

Table S1. Percentage of cell aggregates with and without cavity derived from single blastomeres and control 8-cell embryos containing OCT4-positive and NANOG-positive cells.

Group	Type of aggregates	Nº of aggregates	OCT4+ (n)	NANOG+ (n)
Blastomeres	Cavity	36	86.1 (31)	77.8 (28)
	No cavity	62	90.3 (56)	75.8 (47)
8-cell embryos	Cavity	3	100 (3)	100 (3)
	No cavity	7	100 (7)	100 (7)

Table S2. Mean number of total, OCT4-positive and NANOG-positive cells in cell aggregates with and without cavity derived from single blastomeres and control 8-cell embryos.

Group	Type of aggregates	Total cells	OCT4+ cells	NANOG+ cells
Blastomeres	Cavity	29.9 ± 1.5	6.3 ± 0.8	3.4 ± 0.5
	No cavity	28.7 ± 1.5	6.8 ± 0.8	3.2 ± 0.4
8-cell embryos	Cavity	111.3 ± 7.4	30.7 ± 4.3	16.3 ± 1.8
	No cavity	88.3 ± 13.4	29.0 ± 4.6	16.3 ± 3.0

Table S3. Mean ratios of OCT4+/total cells, NANOG+/total cells and NANOG+/OCT4+ cells in cell aggregates with and without cavity derived from single blastomeres and control 8-cell embryos.

Group	Group	OCT4+/total cells	NANOG+/total cells	NANOG+/OCT4+
Blastomeres	Cavity	0.23 ± 0.03	0.12 ± 0.02	0.57 ± 0.06
	No cavity	0.26 ± 0.03	0.13 ± 0.02	0.48 ± 0.04
8-cell embryos	Cavity	0.28 ± 0.04	0.15 ± 0.03	0.54 ± 0.07
	No cavity	0.33 ± 0.03	0.19 ± 0.02	0.56 ± 0.02
